# Exposure to effluent from pharmaceutical industry induced cytogenotoxicity, hematological and histopathological alterations in *Clarias gariepinus* (Burchell, 1822)

**Published:** 2019-02-06

**Authors:** Chibuisi G. Alimba, Khalid O. Adekoya, Olufemi O. Soyinka

**Affiliations:** 1 Cell Biology and Genetics Unit, Department of Zoology, University of Ibadan, Nigeria; 2 Leibniz Research Centre for Working Environment and Human Factors (IfADo), Technical University of Dortmund, 44139 Dortmund, Germany; 3 Department of Cell Biology and Genetics, University of Lagos, Akoka, Lagos, Nigeria; 4 Department of Marine Sciences, University of Lagos, Akoka, Lagos, Nigeria

**Keywords:** acute toxicity, African catfish, hematology, histopathology, micronucleus assay, untreated pharmaceutical effluent

## Abstract

Pharmaceutical effluents contain toxic xenobiotics capable of contaminating aquatic environments. Untreated effluents are illegally discharged into aquatic environment in most developing countries. Pharmaceutical effluent induced alterations in biomarkers of genetic and systemic damage on rodents. However, information is relatively scarce on the possible cytogenotoxicity and systemic toxicity of this effluent on aquatic vertebrates. The study herein assessed the cytogenotoxic, hematological and histopathological alterations induced by pharmaceutical effluent in *Clarias gariepinus*. 96 h acute toxicity of the effluent was determined after *C. gariepinus* was exposed to six different concentrations (10 - 60 %) of the effluent. Subsequently, fish was exposed to sub-lethal concentrations (2.18 - 17.41 %) obtained from the 96 h LC_50_ for 7 and 14 days after which micronucleus (MN) and nuclear abnormalities (NAs) in peripheral erythrocytes were assessed as cytogenotoxic biomarkers, alterations in hematological indices and histopathological lesions were also examined. Fish, concurrently exposed to dechlorinated tap water and benzene (0.01 mL/L), served as negative and positive controls respectively. The derived 96 h LC_50_ of 17.41 % which was 1.89 times more toxic than the 24 h LC_50_ (32.95 %) showed that the effluent induced concentration-dependent mortality according to exposure duration. The effluent caused significant (*p*<0.05) time-dependent increase in the frequency of MN and abnormal nuclear erythrocytes compared to the negative control. Also, there was decrease in total erythrocyte counts, hemoglobin and hematocrit concentrations and increase in leucocyte and lymphocyte counts. The effluent induced pathological lesions on gills, liver and kidneys of treated fish. Higher physicochemical parameters than standard permissible limits in the effluent are capable of inducing genomic instability and systemic damage in fish. Pharmaceutical effluent can increase micropollutants in aquatic environmental and health risks to aquatic biota. There is need to promulgate stringent laws against illegal discharge of effluents into aquatic environment.

## Introduction

Increasing contamination of the aquatic environment worldwide has been associated with improper discharge of solid wastes, industrial, medical and agricultural effluents (Ibekwe et al., 2012[32]; Larsson, 2014[38]; Alimba et al., 2017[5]). This is raising concern in most countries worldwide, due to the deteriorating effects on surface and underground water and sediment qualities of most aquatic environment, and hence the deleterious health effects on biotic communities (Chigor et al., 2012[14]; Othman et al., 2012[49]). Effluents from pharmaceuticals and personal care products (PPCPs), along with metals and metalloids are increasingly being released directly into aquatic environment via untreated and or poorly treated wastewater and sewage treatment facilities (Nikolaou et al., 2007[45]; Blair et al., 2013[13]; Larsson, 2014[38]). This increase is linked with the quest for quality life expectancy of the rising human and animal populations that depend on pharmaceuticals for their sustenance. 

Occurrence of pharmaceuticals in the aquatic environment has been the research focus for over three decades now (Ternes, 1998[56]; Daughton and Ternes, 1999[19]). There are several reports on the presence of pharmaceuticals and their metabolites in most aquatic environments worldwide (Zuccato et al., 2000[64]; Nikolaou et al., 2007[45]; Kookana et al., 2014[36]). Many of these reports showed that these pharmaceuticals were commonly observed in aquatic environments of most high income nations, with very scanty information from low- and middle-income countries (Kookana et al., 2014[36]). This may be attributed to low environmental awareness or ignorance among developing countries on the occurrence of drugs in aquatic environment. Also inability to afford high standard equipment for analysis of drugs in the aquatic matrix, a common phenomenon in most low- and middle-income nations, may be a contributing factor. However, in recent times, there is an increase in awareness among researchers from Africa and Asia on the need to investigate pharmaceuticals and their metabolites in most surface and underground water bodies. For instance, Agunbiade and Moodley (2016[2]) reported eight acidic pharmaceuticals; four antipyretics (Ibuprofen, Ketoprofen, Diclofenac and Aspirin), three antibiotics (Ampicillin, Ciprofloxacin and Nalidixic acid), and one lipid regulator (Bezafibrate) in wastewater, surface water, and sediments from Msunduzi River in the province of KwaZulu-Natal, South Africa. Also, Olarinmoye et al. (2016[47]) observed thirty seven pharmaceuticals classified as antibiotics, estrogens and lipid-lowering drugs in surface water and industrial, domestic and hospital sewage sludge from Lagos State, Southwest, Nigeria. The presence of these drugs in waterways from low- and middle-income nations was attributed to poor enforcement of regulation and laws restricting indiscriminate discharge of untreated pharmaceutical, hospital and sewage wastewaters directly into the aquatic environment. Also some pharmaceutical industries have shifted from high income countries to low- or middle-income countries mainly due to reduced production costs (Kookana et al., 2014[36]). This is expected to accelerate industrialization and population increase, which will invariably increase wastewater generation and discharge into the environment (Agunbiade and Moodley, 2016[2]).

Pharmaceuticals in aquatic environment occur in concentrations ranging from Limits of Detection (LOD = 8.84 µg/L) to levels exceeding the ecotoxicological predicted no-effect concentrations (PNEC) (Kookana et al., 2014[36]). Most of these pharmaceuticals are active chemicals synthesized to exert specific physiological change(s) on targeted organ(s) of species (mostly mammals). However, they can also affect non-targeted aquatic biota and adversely cause disturbance on the integrity of body systems even at low concentrations (Hernando et al., 2006[28]; Maselli et al., 2015[41]). Pharmaceutical effluents contain mixture of various classes of organic and inorganic micropollutants that are capable of inducing toxicological effects on aquatic organisms. In Nigeria, due to high cost of effluent treatment, most industries illegally discharge untreated effluent directly into aquatic environment. This act is worrisome and has elicited public concern due to increasing occurrence of drugs and metals in coastal waters via Nigerian waterways (Olarinmoye et al., 2016[47]). The individuals and interactions of mixture constituents of the effluents are eliciting adverse impacts on aquatic life (Alimba et al., 2015[7]). Micropollutants in pharmaceutical effluents can cause somatic mutations and systemic toxicity in fish which may cause tumor formation, biodiversity loss and mortality (Viarengo et al., 2007[60]). 

Laboratory designed experiment to simulate the genotoxicity and systemic toxicity of pharmaceutical effluents in aquatic environment using fish is scarce. Available reports have shown that pharmaceuticals elicited acute toxicity on different aquatic invertebrates (Zounková et al., 2007[63]). Pharmaceutical effluent is capable of causing alterations in gene expressions and enzyme activities of plasma vitellogenin which may lead to intersex in fish species (Larsson, 2014[38]). Also, there is evidence that pharmaceutical effluents significantly induced somatic and germ line genotoxicity in rodents (Zhao et al., 2007[62]; Bakare et al., 2009[11]; Adeoye et al., 2015[1]). The use of cytogenetic markers in the routine monitoring of industrial effluents for the presence of xenobiotics is important since DNA damage in aquatic biota may reduce survival by affecting prompt reproduction and increase pollutant-induced stress syndromes (Malins et al., 1988[40]).

The study herein aims at increasing knowledge on the genotoxicity and systemic toxicity induced by untreated pharmaceutical effluent in *Clarias gariepinus*. To achieve this aim, acute toxicity (96 h LC_50_), one of the preliminary toxicological screening bioassay for xenobiotics (OECD, 1992[46]; Akhila et al., 2007[3]), was used to determine the concentration of the pharmaceutical effluent that caused 50 % mortality to the animal model, juvenile *Clarias gariepinus*. Subsequently, the formation of micronucleated (MN) and nuclear abnormalities (NAs) in erythrocytes, alterations in the histological architecture of the liver, gill and kidney, as well as changes in the hematological indices were assessed following sub-chronic exposure to sub-lethal concentrations (96 h LC_50_) of the effluent. 

## Materials and Methods

### Effluent collection, physico-chemical and heavy metals analysis

Pharmaceutical effluent used in the study herein was obtained from a pharmaceutical industry located at Satellite Town, Lagos State, Nigeria. Composite mixture of the untreated effluent was collected into a 25 L transparent high density polyethylene plastic container. The pH was measured and transported in a dark condition using poly-fiber container with cold ice to the animal house unit of the Department of Cell Biology and Genetics, University of Lagos for further processing. The physical and chemical parameters; biochemical oxygen demand (BOD), chemical oxygen demand (COD), dissolved oxygen (DO), turbidity, alkalinity, chlorides, sulphates, nitrates, ammonia and phosphates were analyzed using standard methods (APHA, 2005[9]). Metals and metalloids; arsenic (As), cadmium (Cd), chromium (Cr), lead (Pb), copper (Cu), iron (Fe) and manganese (Mn), were also analyzed in compliance with standard method (APHA, 2005[9]; US EPA, 2014[58]) using Perkin-Elmer A3100 atomic absorption spectrophotometer for the metals. 

### Clarias gariepinus collection and acclimatization for the experimental study

Juvenile *C. gariepinus *with mean ± SD body weight, 12.05 ± 2.30 g and length 9.40 ± 1.50 cm, obtained from Fish farm along Badagry, Lagos State, were used for the study. They were acclimated to laboratory conditions of 26.1 ± 3.0 ^o^C and 12/12 hour dark/light modes for 14 days prior to the experimental set-up. They were stocked at a population density of 10 fish per 25 L aquarium and fed 10 % of their body weight with basal diet containing 35 % crude proteins, rice bran, fish meal and mustard oil-cake (Coppens 2 mm floating feed^®^), twice daily. Guide for care and use of laboratory animals published by the US National Institutes of Health (NIH Publication No. 85-23, revised in 1996) (Gad, 2007[26]; CIOMS, 2012[15]), and approved by the ethical committee, University of Lagos for the use of animals in experimental studies was carefully adhered to during the study.

### Acute toxicity testing and assessment of quantal response (mortality) 

After series of range finding tests, 10 juvenile *C. gariepinus* per group, were randomly distributed into seven experimental groups containing 0.0, 10.0, 20.0, 30.0, 40.0, 50.0 and 60.0 % (v/v; effluent / dechlorinated tap water) for a period of 4 days to determine the 96 h acute toxicity (LC_50_) of the effluent. Safe concentration of the wastewater at 96 h was obtained by multiplying the 96 h LC_50_ by a factor of 0.1 in accordance with EIFAC (1998[21]). Toxicity factor (TF) for 24 hourly relative potency measurements of the effluent were also determined. During the acute toxicity testing, the fish were not fed and the test effluent not renewed (non-renewable static bioassay). Also mortality and behavioral patterns of exposed fish in each experimental group were recorded every 24 h in accordance with the guidelines of the Organization for Economic Cooperation and Development (OECD, 1992[46]). Fish was assumed dead when there was no body or operculum movement, even when prodded with a glass rod. 

### Sub-chronic exposure to sub-lethal concentrations of the 96 h LC_50_ effluent

Ten juvenile *C. gariepinus *were randomly distributed into 25 L tanks containing sub-lethal concentrations: 2.18, 4.35, 8.71 and 17.41 % (v/v, effluent / dechlorinated tap water) (corresponding to 1/8, 1/4, 1/2 and x1 of the 96 h LC_50_ respectively) of the effluent for 14 days. Similar treatment was given to fish in 0.01 mL/L (v/v, Benzene / dechlorinated tap water) and dechlorinated tap water as positive and negative controls respectively. The fish was fed three times daily with 10 % feed per body weight and the water for the treatment and control groups replaced every 48 h to minimize volatilization of less stable components of the effluent so as to maintain concentration of the effluent, and reduce accumulation of metabolic wastes and remains of food particles. On days 7 and 14 of the exposure period, peripheral blood was collected from 5 fish, per sampling time, from each of the treatment and control groups via caudal vein into EDTA bottles for micronucleus assay and hematological analysis. The fish was then sacrificed and dissected, and the gills, liver and kidney collected and fixed in Bouin fluid fixative for 48 h prior to histopathological analysis. 

### Micronucleus, hematological and histological analysis 

Thin smear from an aliquot of the peripheral blood was made on three pre-cleaned slides per fish. They were air dried, fixed in absolute methanol for 30 min, and counterstained with 10 % May-Grunwald and 5 % Giemsa stains in accordance with standard procedure (Alimba et al., 2015[4]; Alimba and Bakare, 2016[6]). Three thousand erythrocytes per fish were scored for MN induction at 1000x magnification. Cells scored for nuclear abnormalities (NAs) include those with small MN that is still connected to the nucleus (nuclear bud, Nbud), cells with two nuclei (binucleated, BN) and cells with vacuoles in their nuclei (vacuolated nuclei, VN). Only cells with intact plasma and nuclear membranes were scored (Alimba et al., 2015[4]; Alimba and Bakare, 2016[6]).

Aliquot of the blood in EDTA bottle was analyzed to determine the concentration of the fish hemogram including Red blood cell (RBC) count, hemoglobin (Hb) content, percentage hematocrit (Ht), mean corpuscle hemoglobin concentration (MCHC), mean corpuscle volume (MCV), mean corpuscle hemoglobin (MCH), platelets, total white blood cell (WBC) count and lymphocytes in the treatment and control groups using automated analyzer (Abbott Hematology Analyzer Cell-Dyn 1700, Abbott Laboratories, Abbott Park, Illinois, USA).

The fixed sections of the gills, liver and kidney from the treated and control fish were dehydrated by passing through ascending order of ethyl alcohol-water concentrations, cleared in xylene and embedded in paraffin wax using rotary microtome. Four μm thick sections of the tissues were prepared on slides, stained with Hematoxylin-Eosin (H&E) and mounted in neutral DPX medium for microscopic examination at x400 magnification by trained pathologist.

### Statistical analysis 

Data from the acute toxicity (mortality) were analyzed using probit analysis with SPSS^TM^ version 17.0, and presented as LC_5_ (lethal concentration that caused 5 % mortality), LC_50_ (lethal concentrations that caused 50 % mortality) and LC_95_ (lethal concentration that caused 95 % mortality) at their corresponding 95 % confidence interval. Frequencies of micronucleated and abnormal nuclear (NA) erythrocytes were presented as mean ± SE (standard error). Significant difference among the various treatment and control groups was determined using One-way ANOVA. Dunnett multiple post hoc test was used to compare the degree of significance (p<0.05) of each treatment group with the negative control.

## Results

### Physicochemical and heavy metal analysis of the effluent

Table 1(Tab. 1) presents the results of the physico-chemical parameters, metals and metalloids analyzed in the effluent. The light yellow coloured effluent which was slightly acidic (pH=6.1) contained high concentrations of the analyzed physicochemical parameters and metals than respective values in the negative control (dechlorinated tap water) and National Environmental Standards and Regulations Enforcement Agency (NESREA, Nigeria) allowable limits for effluent quality criteria standards. However, nitrate and COD were lower than permissible limits but higher than the negative (dechlorinated tap water) control. 

### Acute toxicity (mortality) induced by the effluent to juvenile C. gariepinus 

The toxicity indices (daily LC_50_) obtained from the concentration-mortality data decreased according to increase in exposure duration: 32.95 % (24 h LC_50_) < 26.68 % (48 h LC_50_) < 20.55 % (72 h LC_50_) < 17.41 % (96 h LC_50_). The daily LC_50_ along with the LC_5_ and LC_95_ values for the 24 - 96 h acute toxicity (Table 2(Tab. 2)), showed that the pharmaceutical effluent induced concentration-dependent and exposure related mortality of the juvenile *C.* *gariepinus*. The toxicity factor computed from the 96 h LC_50_ (TF = 1.89) showed that the effluent was highly toxic to *C. gariepinus* (Table 2(Tab. 2)). The safe concentration of the waste-water to the juvenile *C. gariepinus* at 96 h exposure period is 1.74 %.

### Micronucleated and abnormal nuclear erythrocyte formation in C. gariepinus 

**Figure 1:**
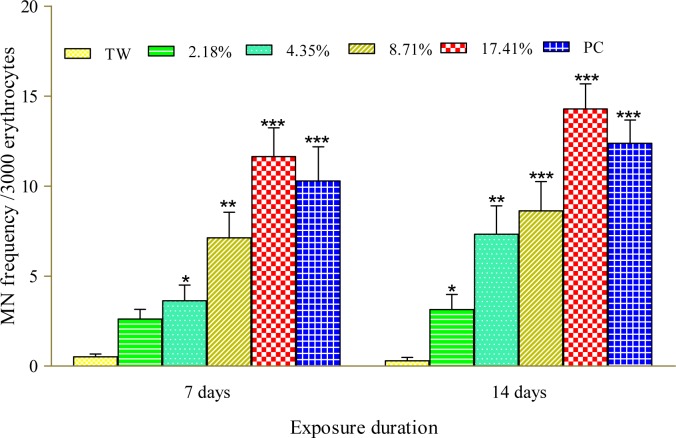
Frequency of micronucleated erythrocytes in *C. gariepinus *exposed to pharmaceutical effluent. ^∗^*p*<0.05; ^∗∗^*p*< 0.01; ^∗∗∗^*p*< 0.001 are significantly different from the negative control (tap water) using Dunnett's multiple post hoc comparison test. PC= Benzene (0.01 mL/L) positive control, TW= tap water (negative control).

**Figure 2:**
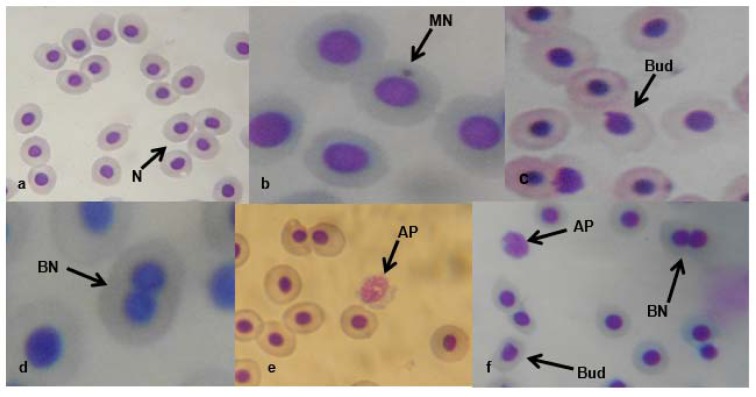
Micronucleated and abnormal nuclear erythrocytes in the effluent treated *C. gariepinus*: (a) normal peripheral erythrocyte (N). (b) Micronucleated erythrocyte (MN). (c) erythrocyte with budded nucleus (Nbud). (d) binucleated erythrocyte (BN). (e) fragmented erythrocyte (AP). (f) AP, BN and Nbud erythrocytes (x1000)

Figure 1(Fig. 1) presents the results of the cytogenetic analysis in *C. gariepinus* exposed to sub-lethal concentrations of the tested effluent. The effluent elicited concentration-dependent significant (*p*<0.05) increase in MN (Figure 2b(Fig. 2)). The induced MN which was also exposure related, showed 4.07, 7.10, 13.96 and 22.82 (corresponding to 2.18, 4.35, 8.71 and 17.41 % concentrations of the effluent respectively) fold increase compared to the negative control during day 7 of exposure, and 10.83, 25.24, 29.72 and 49.28 fold increase than the corresponding negative control during day 14 of exposure. There was significant (*p *< 0.001) increase in the frequencies of nuclear bud (Nbud) (Figure 2c(Fig. 2)), binucleated (BN) erythrocytes (Figure 2d(Fig. 2)), vacuolated (VN) and fragmented nucleus (AP) (Figure 2e(Fig. 2)) formed in the peripheral erythrocytes of effluent exposed fish compared to the negative control. The induced NAs which was concentration-dependent and exposure duration related was in the order; Nbud > BN > VN > AP (Table 3(Tab. 3)). 

### Hematological profile of Clarias gariepinus exposed to pharmaceutical effluent

The sub-lethal concentrations of the pharmaceutical effluent significantly reduced red blood cells (RBC), percentage hematocrit (HCT), hemoglobin (HGB) concentration, but increased white blood cells (WBC) and lymphocytes (except at 2.18 % concentration of the effluent where there was insignificant decrease in lymphocytes compared to the negative control) in a concentration-dependent pattern during 7 day exposure. However, the mean corpuscular volume (MCV), mean corpuscular hemoglobin (MCH) and mean corpuscular hemoglobin concentration (MCHC) were insignificantly (*p *> 0.05) increased in the treated fish compared to the negative control during 7 day exposure. For 14 day exposure period, the tested effluent significantly (*p *< 0.05) reduced red blood cells (RBC), percentage hematocrit (HCT), hemoglobin (HGB) concentration, but increased white blood cells (WBC) (except at the 2.18 % concentration of the effluent where there was insignificant decrease in the leucocyte counts compared to the negative control) and lymphocytes. The altered hematological indices in the exposed fish were not concentration-dependent. The mean corpuscular volume (MCV), mean corpuscular hemoglobin (MCH) and mean corpuscular hemoglobin concentration (MCHC) data from fish exposed for 14 days were insignificantly (*p *> 0.05) increased compared to the negative control and in a concentration-independent pattern (Table 4(Tab. 4)). 

### Histological alterations in tissues of C. gariepinus exposed to the effluent

**Figure 3:**
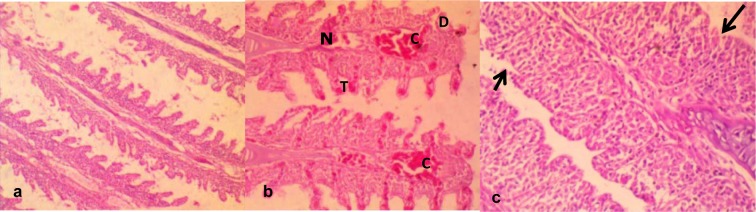
a. Gill from a control group showing apparently normal gill filament and gill lamellae. b. There is severe congestion (C) of the blood capillaries; necrosis (N) thickening (T) of the gill filament, disorganization of the gill lamella. c. Loss of gill lamellae (arrow); the covering epithelium of the operculum is markedly separated from the central cartilaginous core by sparse amounts of loose connective tissues. Mag. x400

Gills collected from fish exposed to dechlorinated tap water (negative control) presented apparently normal filaments and lamellae (Figure 3a(Fig. 3)). However, gills from fish exposed to sub-lethal concentrations of the effluents for 7 and 14 days revealed some histopathological lesions which include severe congestion of the blood capillaries and thickening of the filaments (Figure 3b(Fig. 3)). Also gill lamellae were absent from the sections of some fish from the treatment groups, and the covering epithelium of the operculum markedly separated from the central cartilaginous core by sparse amounts of loose connective tissues (Figure 3c(Fig. 3)).

**Figure 4:**
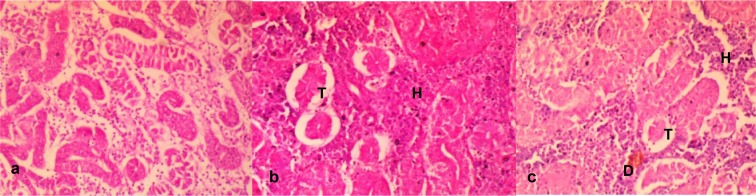
a. Sections of the kidney from the control fish showing apparently normal tubular and hematopoietic compartment. The tubular is a closely packed tubule with glomeruli. b. Section of kidney from effluent treated fish showing severe depletion of the tubular (T) and hematopoietic (H) compartments thus appearing more prominent. Mag. x400 c. Section from the effluent treated fish showing tubules that are widely separated from each other with decrease in the tubular (T) compartment and accompanying increase in the hematopoietic (H) compartment; there are multiple foci of degenerated tubules (D). Mag. x400

Kidney sections from fish exposed to tap water (negative control) showed apparently normal tubular (TC) and hematopoietic compartment (HC), with the TC consisting of closely packed blood capillaries and glomeruli (Figure 4a(Fig. 4)). There were multiple foci of tubular degeneration and severe depletion of the TC and HC in the effluent treated fish (Figure 4b-c(Fig. 4)). 

**Figure 5:**
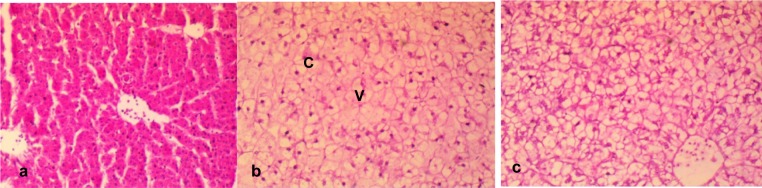
a. Section of the liver of a negative control fish showing closely packed hepatic plates with the hepatocytes not showing visible cytoplasmic vacuoles and with intact bile ducts. b. The hepatocytes of effluent treated fish contain multiple moderate-sized vacuoles (V), large prominent bile duct with a moderately congested (C) of the central veins and disarrayed hepatocytes. c. In the effluent treated fish, there are widespread multiple foci of hepatocytes with clear cytoplasmic vacuoles. Also there are widespread large multiple cytoplasmic vacuoles within the hepatocytes, and the central veins moderately congested with disarrayed hepatocytes. Mag. x400.

The histological presentation of the hepatic sections of the tap water treated fish showed the normal architecture of fish hepatocytes with closely packed hepatic plates without cytoplasmic vacuoles (Figure 5a(Fig. 5)). However, some histological lesions including congestions of central veins, large prominent bile ducts and multiple moderate-sized vacuoles were observed in the liver tissue of the effluent treated fish (Figure 5b and 5c(Fig. 5)).

## Discussion

Increasing utilization of pharmaceuticals in order to improve quality of health and extend life expectancy in humans and domesticated animals has an inevitable consequence of increasing surface and ground water contamination with biologically active chemicals including toxic metals (Corcoran et al., 2010[16]; Olarinmoye et al., 2016[47]). Despite the possible adverse effects this may pose on aquatic wildlife, studies are scanty that have characterized the possible health effects of pharmaceutical effluents on aquatic vertebrates (including fish). 

The 96 h LC_50_ (17.41 %) which was 1.89 (TF=1.89) times more toxic than the 24 h LC_50_ (32.95 %) showed that the effluent was highly toxic to the juvenile stage of *C. gariepinus*. The induced mortality was concentration-dependent and exposure duration related. In comparison with previous studies from our laboratory where similar sized juvenile *C. gariepinus* served as bio-indicator, the 96 h LC_50_ obtained for Aba Eku (43.29 %) and Olusosun (34.52 %) landfill leachates (Alimba and Bakare, 2016[6]) showed that the tested effluent herein was more toxic to the fish than solid waste landfill leachates, but was less toxic than abattoir effluent (96 h LC_50_ = 6.28 %) (Alimba et al., 2015[7]), textile effluent (96 h LC_50_ = 8.02 %) (Ayoola et al., 2012[10]) and hospital effluent (96 h LC_50_ = 1.30 %) (Alimba et al., 2017[5]). This observation showed that juvenile *C. gariepinus* responded differently to mortality and deleterious effects elicited by different industrial effluents. Hence, it can be concluded that *C. gariepinus* is a suitable bio-indicator for monitoring the toxicity of effluents. The derived safe concentration of 1.74 % of the effluent for the juvenile *C. gariepinus* used herein is significantly low compared to the volume of untreated wastewater that is directly discharged into aquatic environment in most low- and middle-income nations (Sogbanmu et al., 2016[55]). This may suggest a higher level of morbidity and eventual death of fish and many other lower vertebrates that inhabit such wastewater contaminated aquatic environment. The analyzed physicochemical parameters, toxic metals and metalloids (Table 1(Tab. 1)), in the effluent possibly interacted synergistically, antagonistically or additively to induce mortality in the fish (Magdaleno et al., 2014[39]). High total dissolved solids analyzed in the effluent may be due to inert solids and particulate matters. These solids are capable of clogging the gill system and may suffocate the fish to death (FAO, 1991[23]). 

The selected sub-lethal concentrations of the tested pharmaceutical effluent did not lead to immediate mortality of the fish, however it elicited alterations in the biomarkers of somatic mutations and systemic damage examined. MN assay, which has been used for over 30 years as biomarker of cytogenetic damage in fish (Al-Sabti, 1986[8]), due to its reliability, sensitivity and cost effectiveness, showed that the tested effluent is cytogenotoxic. Significant increase in micronucleated erythrocytes in the effluent treated *C. gariepinus* indicated that the constituents of the effluent are clastogenic and/or aneugenic to fish genetic materials. The observed genome instability elicited by the tested effluents in *C. gariepinus* herein, had been previously reported in mice and rat wherein pharmaceutical effluents increased frequency of chromosome aberrations and micronucleated polychromatic erythrocytes in bone marrow erythrocytes of mice and rats (Bakare et al., 2009[11]; Adeoye et al., 2015[1]), and increased germ line mutation in mice (Zhao et al., 2007[62]; Bakare et al., 2009[11]). Scoring NAs along with MN in *C. gariepinus* exposed to mixture of xenobiotics in leachates and effluents is an efficient and reliable biomarker of cytogenetic damage (Ayoola et al., 2012[10]; Bakare et al., 2013[12]; Alimba et al., 2015[7][4], 2017[5]; Alimba and Bakare, 2016[6]). Significant increase in total nuclear abnormalities in the effluent exposed *C. gariepinus* compared with the negative control suggests that the constituents of the effluent induced perturbation in cell cycle and DNA synthesis during hematopoiesis in the fish (Udroiu, 2006[57]). Nuclear bud formation is associated with the entrapment of extra-chromosomally amplified DNA during S-phase and is related to genotoxicity events (Fenech et al., 2011[25]). Binucleated cells (biomarker of cytotoxicity) are formed possibly due to the presence of cytotoxins in the effluents which altered cytokinesis during M phase of the cell cycle. Increased fragmented (apoptotic) and vacuolated nuclei observed in the erythrocytes of effluent exposed *C. gariepinus* may be linked to alterations in p53 protein expression which led to the activation of antioxidant genes associated with apoptotic cell formation (Verlhac and Gabaudan, 1994[59]), and or damaged erythrocytes during hematopoitic process, which were eliminated by programmed cell death (apoptosis) (Pulido and Parrish, 2003[50]). There is paucity of information on the possible cytogenotoxic effects of pharmaceutical effluents on aquatic vertebrate including fish. However, it is important to note that significant increase in frequencies of MN and NAs are associated with genome instability which has been strongly correlated with genetic related syndromes; different pathogenesis, reproductive dysfunctions and cancer formation in fish (Malins et al., 1988[40]; Kurelec, 1993[37]; Alimba et al., 2015[7]; Daiwile et al., 2015[18]). The analyzed metals (Table 1(Tab. 1)) in the effluent are potent genotoxins in both *in vivo* and *in vitro* test systems and may account for the observed MN and NAs induction in the effluent exposed fish (Jiraungkoorskul et al., 2008[34]; Guedenon et al., 2015[27]). 

Considering the importance of blood during body and systemic circulation in fish, alteration in hematological indices in response to water contamination is considered a sensitive biomarker of fish health (Seriani et al., 2015[51]). Usually changes in hematological indices appear first before the onset of any morphological and degenerative damage in fish (Mazon et al., 2002[42]). Decrease in RBC, hemoglobin concentrations and percentage hematocrit in *C. gariepinus* during both 7 and 14 day exposures to the effluent suggests anemic condition in the exposed fish. This may be due to the deleterious effects of the effluent constituents on the hemotopoietic system (mainly the kidney system) by inhibiting erythropoiesis via transferrin dysfunction (Javed et al., 2016[33]). Increase in MCV, MCH and MCHC in the effluent exposed fish, although not statistically significant, may suggest that the anemic condition induced by the effluent is macrocytic hyperchromic anemia. It is possible that the critical hematotoxic conditions on the fish are in tandem with the high mortality observed in the acute toxicity (Table 2(Tab. 2)). Macrocytic hyperchromic anemia has been associated with defects in DNA synthesis during erythropoiesis (Hoffman et al., 2009[30]). Total white blood cell (WBC) and differentials in fish form components of the non-specific immune cells (da Silva Correa et al., 2016[17]). Leukocytosis and lymphocytosis observed herein may indicate physiological and immunological (inflammatory) challenges in response to the toxic effects of the effluent (Duthie and Tort, 1985[20]). Leukocytosis is directly related to severity of damage and stress induced by metals with a consequential result of immunological defense stimulation (Javed et al., 2016[33]). Similar trends of alteration in hematological indices were reported in *Cyprinus carpio* exposed to Cr(VI) (Shaheen and Akhtar, 2012[52]), in *Labeo rohita* exposed to effluents from paint, dye and petroleum industries (Zutshi et al., 2010[65]), in *Oreochromis mossambicus* exposed to Cd (Shalaby, 2001[53]) and in rats exposed to pharmaceutical effluent for 28 days (Adeoye et al., 2015[1]). These may be linked to individual and/or interactive effects of the effluent constituents on the metabolic and hemotopoietic activities in the fish.

Changes in the histology of tissues in fish have been widely used, both in laboratory regulated experiments (Mela et al., 2007[43]) and field studies (Alimba et al., 2015[7]) to assess fish health. Histopathology, as a biomarker of toxicity, is relevant in monitoring organ specific damage induced by environmental pollutants and relates the damage organs to specific physiological function in the fish body (Evans, 1987[22]). Furthermore, histopathological findings corroborate functional biomarkers to provide specific information on the acute and chronic effects of toxicants on targeted organs (Evans, 1987[22]; Mela et al., 2007[43]). 

Gill epithelium of teleost, apart from being the gaseous exchange site, is also responsible for ionic regulation, acid-base balance, and nitrogenous waste excretion in fishes (Hoar and Randall, 1984[29]). The observed severe congestion of the blood capillaries, necrosis and thickening of the gill filament and disorganization of the lamellae in effluent exposed *C. gariepinus* (Figure 3b and 3c(Fig. 3)) showed that constituents of the effluent caused structural alterations on the gills. The effluent constituents come in direct contact with the gills and considering that the gill filaments and lamellae have increase surface area of exposure to contaminants, makes gills the most critical site of toxicity (Evans, 1987[22]; Wood et al., 2002[61]). Also considering that the gills are highly metabolically active, they are readily prone to damage from environmental micropollutants, mostly the toxic metals via oxidative stress induction (Farombi et al., 2007[24]). The observed histopathological lesions in the effluent exposed *C. gariepinus* had been similarly reported in *Oreochromis niloticus* which were exposed to petroleum refinery effluent (Onwumere and Oladimeji, 1990[48]) and in free ranging *Synodontis clarias* caught from toxic metal and organic compounds polluted Lekki Lagoon and Ogun River in Nigeria (Alimba et al., 2015[7]).

It is well known that the liver and kidneys are among the major organs that readily respond to toxic effects of a wide variety of environmental micropollutants owning to their involvement in absorption and bio-concentration of pollutants (Hook, 1980[31]; Koca et al., 2008[35]). The liver is mainly involved in metabolism, detoxification, storage, and excretion of xenobiotics and their metabolites, while the kidneys serve as the main hematopoietic organ of most teleost (Mela et al., 2007[43]). These functions make them targets for xenobiotics induced pathophysiological injuries (Mela et al., 2007[43]; Koca et al., 2008[35]; Alimba et al., 2015[7]). That these organs readily bio-accumulate toxic metals and metabolize organic toxicants (Silva and Martinez, 2007[54]), may account for the pathological lesions observed in the treated fish compared to the negative control. The effluent induced hepatic and renal damage to the exposed fish. The observed lesions may be attributed to the direct or indirect deleterious actions of the effluent constituents on the tissues following the attempt by the fish to detoxify and eliminate the toxic metals and other xenobiotics from the body. The observed pathological lesions in the exposed fish herein have been similarly reported in rats orally exposed to pharmaceutical effluent for 28 days (Adeoye et al., 2015[1]). This suggests that the constituents of untreated pharmaceutical effluent pose great health challenge to both terrestrial and aquatic biota. Multiple foci of degenerated tubules and severe depletion of the hematopoietic compartment of the kidney, the organ of blood production in teleost, corroborate the observed alterations in the fish hemogram and micronucleus and abnormal nuclear formation in the erythrocytes. 

The findings herein showed that pharmaceutical effluent contains varying concentrations of toxic metals, metalloids and physicochemical parameters higher than standard permissible limits. The effluent increased frequency of MN and NAs in erythrocytes, altered hematological indices and induced histopathological lesions in gills, liver and kidney in *C. gariepinus*. This suggests the constituents of pharmaceutical effluents as emerging carcinogens and mutagens that are capable of increasing genome instability, altering blood cell indices and causing pathological lesions in fish tissues. This is a threat to the functioning aquatic ecosystems and the survival of aquatic biota.

## Acknowledgement

The authors appreciate Angela I. Chikeluba for her technical assistance during the study.

## Funding

The authors declare that the study herein was not funded by any funding body.

## Conflict of interest

The authors declare that no form of conflict of interest exists.

## Figures and Tables

**Table 1:**
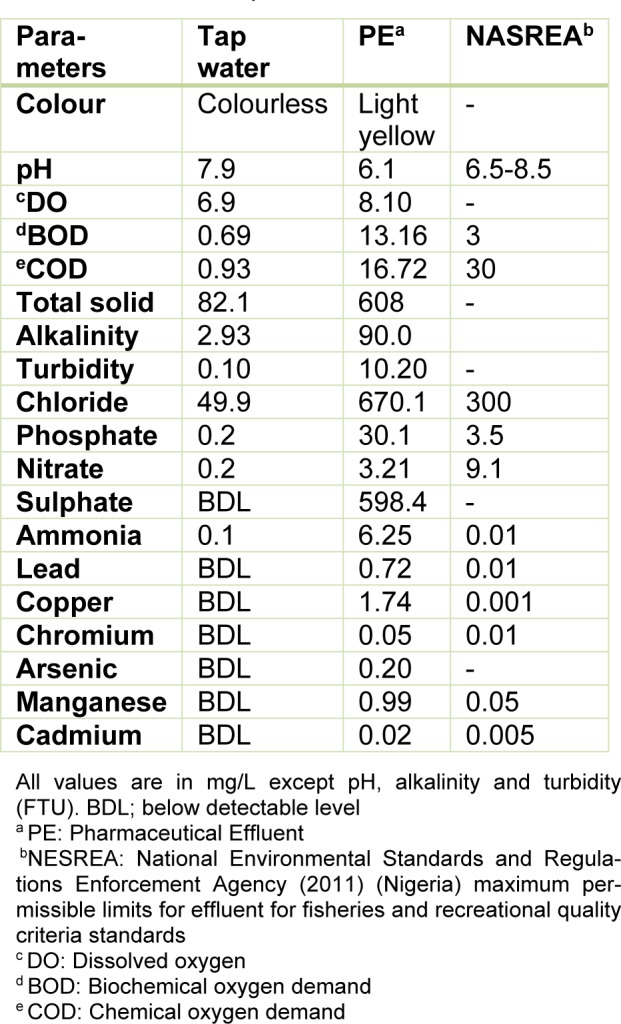
Physicochemical parameter and metal analyses of the pharmaceutical effluent (PE), tap water and national permissible standards

**Table 2:**
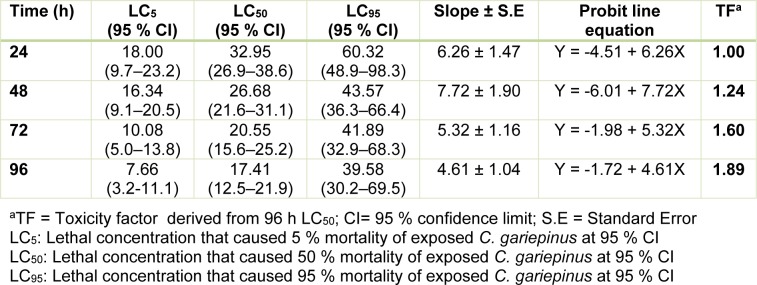
96 h acute toxicity determination of pharmaceutical effluent (PE) using *Clarias gariepinus*

**Table 3:**
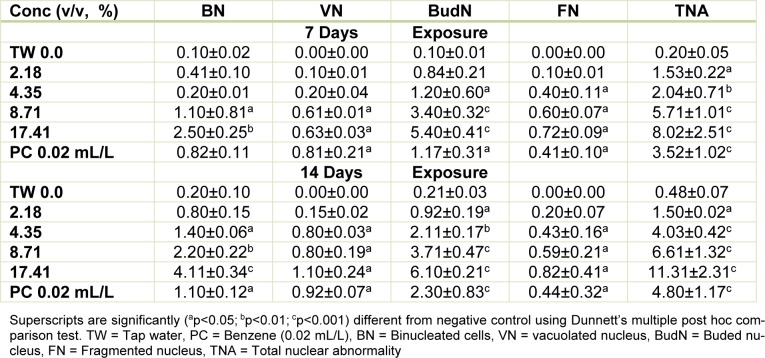
Mean (± SE) of nuclear abnormalities (NAs)/3000 peripheral erythrocytes of *C. gariepinus* exposed to pharmaceutical effluent for 7 and 14 days

**Table 4:**
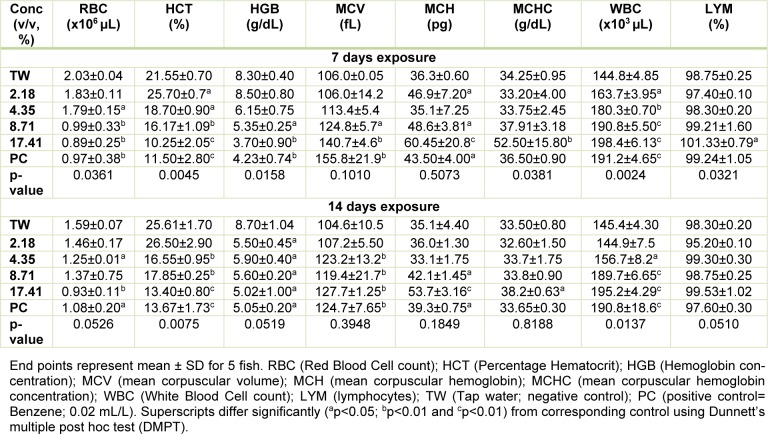
Hematological profile of *C. gariepinus* exposed to the pharmaceutical effluent for 7 and 14 days
